# CD28 Costimulation of T Helper 1 Cells Enhances Cytokine Release *In Vivo*

**DOI:** 10.3389/fimmu.2018.01060

**Published:** 2018-05-16

**Authors:** Daniela Langenhorst, Stephanie Haack, Selina Göb, Anna Uri, Fred Lühder, Bernard Vanhove, Thomas Hünig, Niklas Beyersdorf

**Affiliations:** ^1^Institute for Virology and Immunobiology, University of Würzburg, Würzburg, Germany; ^2^Institute for Multiple Sclerosis Research and Neuroimmunology, University Medical Centre Göttingen, Göttingen, Germany; ^3^Centre de Recherche en Transplantation et Immunologie UMR 1064, INSERM, Université de Nantes, Nantes, France; ^4^Institut de Transplantation Urologie Néphrologie (ITUN), CHU Nantes, Nantes, France; ^5^OSE Immunotherapeutics S.A., Nantes, France

**Keywords:** CD4^+^ T helper cells, T helper 1 cells, antigenic recall, CD28 costimulation, cytokine secretion, mouse, human

## Abstract

Compared to naive T cells, differentiated T cells are thought to be less dependent on CD28 costimulation for full activation. To revisit the role of CD28 costimulation in mouse T cell recall responses, we adoptively transferred *in vitro* generated OT-II T helper (Th) 1 cells into C57BL/6 mice (Thy1.2^+^) and then either blocked CD28–ligand interactions with Fab fragments of the anti-CD28 monoclonal antibody (mAb) E18 or deleted CD28 expression using inducible CD28 knock-out OT-II mice as T cell donors. After injection of ovalbumin protein in adjuvant into the recipient mice we observed that systemic interferon (IFN)γ release strongly depended on CD28 costimulation of the Th1 cells, while secondary clonal expansion was not reduced in the absence of CD28 costimulation. For human memory CD4^+^ T cell responses we also noted that cytokine release was reduced upon inhibition of CD28 costimulation. Together, our data highlight the so far underestimated role of CD28 costimulation for the reactivation of fully differentiated CD4^+^ T cells.

## Introduction

For full activation, naive T cells require at least two signals: signal one originating from the interaction of the T cell receptor (TCR) with peptide/major histocompatibility complexes and the second signal stemming from the interaction of CD28 with its ligands CD86 and CD80 on antigen-presenting cells (APCs) (1). While the role of CD28 costimulation for naive T cell activation and CD4^+^ Foxp3^+^ regulatory T cell homeostasis and function is very well established (1) less is known about its impact on secondary responses of CD4^+^ T helper (Th) cells. In fact, early experimental evidence indicated that memory T cells might be less dependent on CD28 costimulation than naive T cells (2). A study following up on the requirement of memory CD4^+^ T cells for CD28 costimulation *in vivo* using a mixed population of memory T cells containing about 25% interferon γ (IFNγ)^+^ T helper 1 (Th1) cells came to the opposite conclusion (3). However, in this study CTLA-4-Ig was used to block interactions of CD28 with its ligands. Binding of CTLA-4-Ig to the T cells, which express CD86 and CD80 themselves (4), and induction of indoleamine 2,3-dioxygenase (IDO) expression in APCs (5) hamper the interpretation of these data. Another recent and elegant study addressed the role of CD28 in effector/memory CD4^+^ T cell responses by using OX40-Cre floxed CD28 mice leading to CD28 deletion after initial antigen recognition, i.e., within the first 48 h of the primary immune response *in vivo* (6). Under these conditions, CD28 costimulation was not only required for Th1 cell expansion, but also for the differentiation and maintenance of T follicular helper cells (6). OX40-Cre-induced CD28 deletion does, however, not fully reflect the situation in humans in whom memory CD4^+^ T cell responses are often triggered years after the first vaccination or first encountered with pathogen-derived antigens. Therefore, we set up our study to analyze the contribution of CD28 costimulation during antigenic recall responses of already differentiated mouse Th1 cells. To this end, we first differentiated ovalbumin (OVA) peptide-specific TCR-transgenic OT-II T cells into Th1 cells *in vitro* before adoptive transfer *in vivo* and induction of genetic deletion of CD28 or antibody-mediated blocking of the interaction of CD28 with its ligands. As both mouse and human polarized CD4^+^ Th cells have been shown to undergo reprogramming under certain conditions *in vitro* and *in vivo* (7–9), we also followed the impact of CD28 costimulation on Th cell lineage stability.

In humans, selective inhibitors of CD28–ligand interactions, i.e., Fab fragments of the anti-CD28 monoclonal antibody (mAb) CD28.3, allow to interrogate the contribution of CD28 costimulation to human memory T cell responses. Blockade of CD28 costimulation with the CD28.3-Fab-derived drug FR104 on a mixed population of CD4^+^ and CD8^+^ human memory (CD45RA^−^ CCR7^−^) T cells has revealed that both alloantigen- as well as virus peptide-driven proliferation of memory T cells is enhanced by CD28 costimulation (10, 11). As our data obtained with mouse OT-II T cells indicated that CD28 costimulation enhanced IFNγ secretion by restimulated Th1 cells, we also studied cytokine secretion by human peripheral blood mononuclear cells (PBMC) upon addition of T cell recall antigens *in vitro*. As for the mouse T cells, CD28 costimulation of human T cells, too, increased cytokine secretion upon antigenic recall.

## Materials and Methods

### Mice

C57BL/6J.Thy1.1^+/−^ (12), C57BL/6J.OT-II Thy1.1^+/+^, C57BL/6J.OT-II Thy1.1^+/+^ ERCre^+/−^ CD28^flox/flox^ inducible CD28 knockout mice and their C57BL/6J.OT-II Thy1.1^+/+^ ERCre^+/−^ CD28^wt/wt^ WT littermates were bred and maintained in the specific pathogen-free animal facility of the Institute for Virology and Immunobiology at the University of Würzburg. To obtain these mouse strains, we used C57BL/6J.OT-II (13) and C57BL/6J.ERCre^+/−^ CD28^flox/flox^ inducible CD28 knock-out mice (14, 15) for crossings. Animals used for experiments were between 6 and 15 weeks old.

### Peripheral Blood Mononuclear Cells

Human PBMCs were prepared from healthy blood donors as a byproduct of platelet concentrates obtained with leukoreduction system chambers [LRS-C; Gambro Trima Accel aphaeresis apparatus, Pall Corp. (16)], diluted in versene, isolated by density gradient centrifugation with Lymphocyte Separation Medium (PAA Laboratories), and washed with ice-cold balanced salt solution (BSS)/0.2% BSA. The leukoreduction chambers were provided anonymously by the Department of Transfusion Medicine of the University Hospital Würzburg in accordance with the guidelines of the Ethics Committee of the Medical Faculty of the University of Würzburg.

### Generation of Th1 Cells and *In Vitro* Conversion (Mouse)

Naïve MACS-sorted CD4^+^CD25^−^ OT-II T cells from spleen and lymph nodes were cultured in RPMI 1640 with l-glutamine, nonessential amino acids, β-mercaptoethanol, sodium pyruvate, penicillin/streptomycin, and 10% FCS (all Gibco) in the presence of Thy1.2 (T cell)-depleted APCs and 2 µM OVA_327–339_ (Charité Berlin). For Th1 differentiation 10 µg/ml anti-interleukin (IL)-4 (11B11, Bio X Cell) and 10 ng/ml IL-12 (R&D Systems) were added—similar to what has been previously described (8). Cell cultures were split on days 2 and 4. For *in vitro* conversion experiments differentiated Th1 cells were washed with BSS/BSA on day 6 and reactivated with fresh T cell-depleted APCs and, for Th0 conditions, with 0.1 µM recombinant human (rh)IL-2 (Proleukin^®^, Novartis); for Th2 conditions—again close to a published protocol (8)—with 10 µg/ml anti-IL-12 (C17.8, Bio X Cell), 10 µg/ml anti-IFNγ (XGM1.2, Bio X Cell), 100 ng/ml recombinant mouse IL-4 (Miltenyi Biotec) and, in addition, 0.1 µM rhIL-2 in the presence and absence of 1 µM OVA_327–339_ and 10 µg/ml Fab fragment of anti-CD28 mAb E18 (Exbio). On days 5 and 10 of the culture we analyzed the cells by FACS.

### *In Vitro* Recall Responses (Human)

Isolated carboxyfluorescein succinimidyl ester diacetate (CFSE) (5 µM) labeled PBMCs were cultured in RPMI 1640 medium supplemented with l-glutamine (Invitrogen), nonessential amino acids (Invitrogen), HEPES (Applichem), β-mercaptoethanol (Invitrogen), sodium pyruvate (Invitrogen), penicillin/streptomycin, and 10% heat-inactivated human AB serum (Sigma-Aldrich) in the presence or absence of 0.1 µg/ml anti-CD3 mAb (HIT3a), 10 µg/ml purified protein derivative (PPD) (Pharmore), 100 mU/ml tetanus and diphtheria toxoid (Td)-RIX (GlaxoSmithKline), and 0.3 µg/ml Fab fragment of the anti-human CD28 mAb CD28.3. To generate Th1 conditions, 1 µg/ml anti-human IL-4 (R&D Systems), 2 ng/ml rhIL-12 (Sigma) and, additionally, 0.1 µM rhIL-2 (Proleukin^®^, Novartis) were added (7). Th2 conditions consisted of 2 µg/ml anti-human IL-12 (R&D Systems), 2 ng/ml rhIL-4 (Miltenyi) and, in addition, 0.1 µM rhIL-2 (7). For Th0 conditions, no further cytokines or antibodies were added. After 6 days of culture the cells were analyzed by FACS.

In some experiments (Figure 5), PBMCs were first stained with anti-CD4 (OKT4), CD45RA (HI100), and CCR7 (G043H7) (all BioLegend) and either CD4^+^ CD45RA^−^ memory T cells or CD4^+^ CD45RA^+^ CCR7^+^ naive T cells were separated from the PBMC by flow cytometric cell sorting. The sorted PBMCs were then also labeled with CFSE and either stimulated alone or in the presence of the previously separated CD4^+^ T cell subset added back before initiation of the cultures. To determine proliferation of CFSE-labeled and unlabeled cells, the cultured PBMCs were stained extracellularly for CD4 and intracellularly for Ki-67 expression (B56, BD) after 4 days of incubation.

### *In Vivo* Recall Responses and CD28 Blockade/Deletion

2 × 10^6^ activated, OVA-specific Th1 cells (day 4 *in vitro*) of OT-II Thy1.1^+/+^, OT-II iCD28ko (OT-II Thy1.1^+/+^ ERCre^+/−^ CD28^flox/flox^) mice or WT littermates were transferred intravenously into C57BL/6 Thy1.1^+/−^ recipients on day 0. 100 µg Fab fragment of anti-mouse CD28 mAb E18 (Exbio) or control antibody MOPC-21 (Bio X Cell) were injected i.p. on five consecutive days, starting with the day after T cell transfer. To delete CD28 expression on iCD28ko donor T cells, 1.25 mg tamoxifen (Hexal AG) was administered in watery solution to recipient mice by oral gavage for four consecutive days, beginning with the day after T cell transfer. Either on day 3 (E18-Fab) or day 9 after T cell transfer (tamoxifen) we injected 10 µg OVA protein (Sigma) in 50 µl phosphate-buffered saline (PBS) emulsified in 150 µl Alum (Serva) s.c. 6, 24, and 48 h after antigen administration blood samples were taken from the tail vein and sera were stored at −80°C until analysis. Lymph node and spleen cells were analyzed 7 days after antigen challenge.

### Flow Cytometry

The following antibodies and dyes were used for FACS analysis of human cells: CD4 (RPA-T4), GATA-3 (16E10A23), T-bet (4B10) (all BioLegend), and dead cell marker Viability Dye eFluor™ 780 (eBioscience). The following antibodies and dyes were used for FACS analysis of mouse cells: CD4 (RM4-5), Thy1.1 (Ox-7), Thy1.2 (30-H12), Gata-3 (16E10A23), IL-4 (11B11), IFNγ (XGM1.2), T-bet (eBio4B10) (all BioLegend), and dead cell marker Viability Dye eFluor™ 780 (eBioscience). For intracellular cytokine analysis, cells were restimulated with 5 ng/ml PMA and 500 ng/ml ionomycin for 4 h. 10 µg/ml BrefA were added after 2 h. Stainings were performed with up to 10^6^ cells from PBMC, lymph node, or erythrocyte depleted spleen cells, in 50 µl of FACS buffer (PBS/0.1% bovine serum albumin/0.02% NaN_3_). After surface staining (30 min, 4°C), cells were fixed for 30 min at 4°C (fixation buffer, eBioscience), permeabilized (permeabilization buffer, eBioscience), and intracellularly stained for Gata-3 and T-bet or IL-4 and IFNγ expression for 45 min at room temperature. The cells were analyzed on a BD™ LSR II flow cytometer with the use of FACS Diva software (all Becton Dickinson). For further analyses of the data, FlowJo (TreeStar Inc.) software was used. Median fluorescence intensity (MFI) ratios of T-bet and Gata-3 were calculated by dividing the median fluorescence intensities of the two markers.

### Analysis of Cytokine Concentrations in Serum and Culture Supernatant

Cytokine concentrations in serum and culture supernatant (mouse and human) were analyzed using the LEGENDplex bead-based immunoassay (BioLegend) according to the manufacturer’s instructions.

### Statistical Analysis

Data are presented as mean + SD or median + range as indicated. Statistical significance was analyzed by two-tailed unpaired *t*-test, one-tailed paired *t*-test, or Mann–Whitney *U* test using GraphPad Prism Software. Values of *p* < 0.05 were considered to be statistically significant.

### Ethics Statement

All animal experiments were performed in accordance with German law and approved by the Regierung von Unterfranken as the responsible authority. The ethics committee of the medical faculty of the University of Würzburg approved the anonymous use of human PBMC from healthy blood donors for this study.

## Results

### IFNγ Release Upon Antigenic Challenge of Th1 Cells *In Vivo* Requires CD28 Costimulation

**Figure 1:**
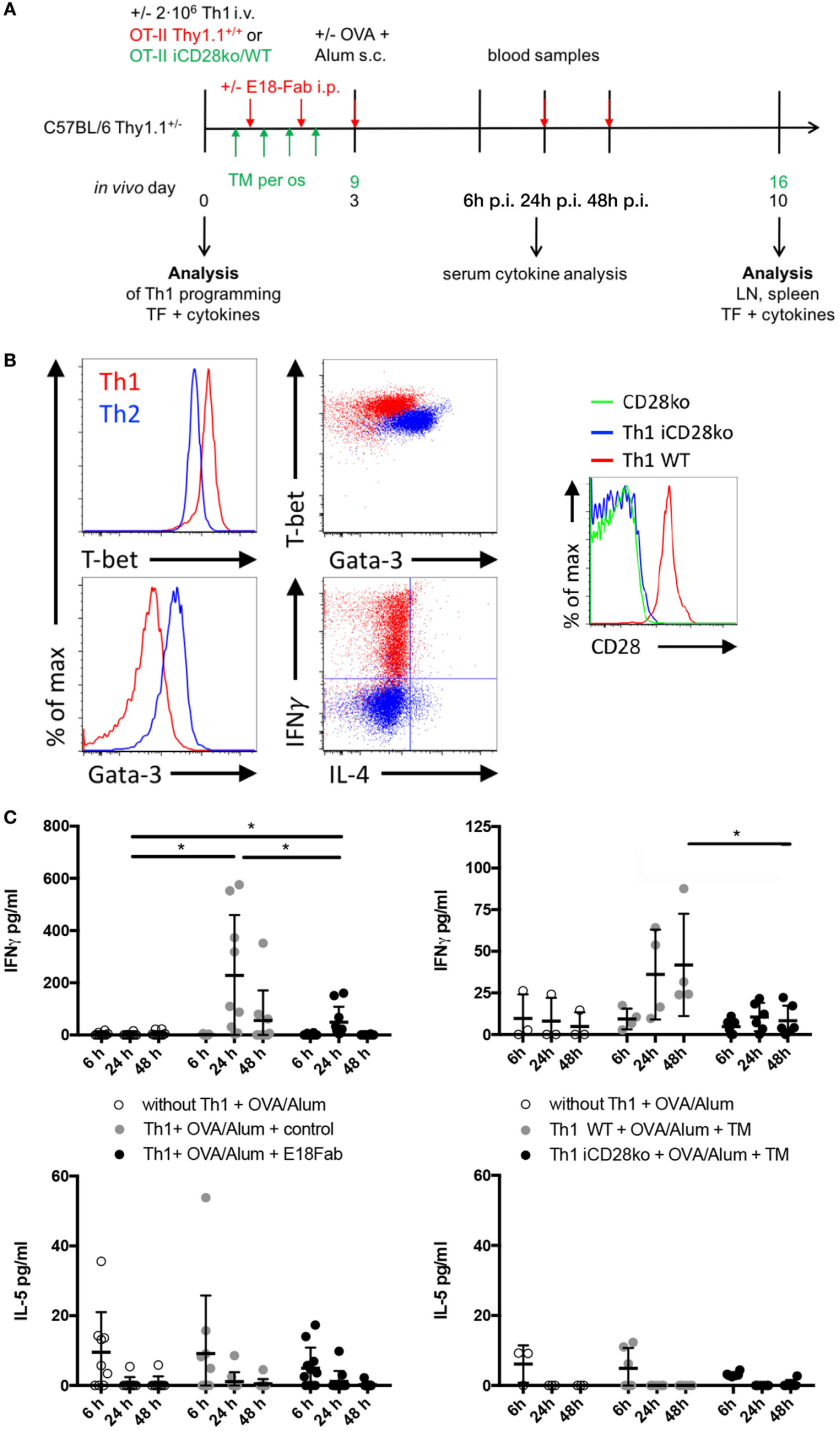
CD28 costimulation of Th1 (T helper 1) cells enhances systemic interferon γ (IFNγ) release, but does not modulate Th1 cell expansion *in vivo*. **(A)** 2 × 10^6^ *in vitro* differentiated Th1 cells of OT-II Thy1.1^+/+^ mice, OT-II inducible CD28 knock-out (iCD28ko) mice or WT littermates were transferred into Thy1.1^+/−^ mice. For CD28 blockade, E18-Fab or control was injected on the following 5 days. CD28 deletion was induced by tamoxifen treatment from days 1 to 4 *in vivo*. 3 days and 9 days after T cell transfer, respectively, recipient mice received ovalbumin/Alum by subcutaneous administration. Serum samples were taken 6, 24, and 48 h later. Lymph node and spleen cells were analyzed 7 days after antigen challenge. **(B)** T-bet, Gata-3, IFNγ, and interleukin (IL)-4 expression in OT-II Th1 (red) and, for comparison, Th2 cells (blue) after 5 days of differentiation and CD28 expression by OT-II iCD28ko Th1 cells 7 days after transfer (histogram). **(C)** Serum cytokine concentration of IFNγ and IL-5 after E18-Fab-mediated CD28 blockade (left figures) and inducible CD28 deletion (right figures). Data for individual mice are shown together with mean and SD. Results were pooled from three to five independent experiments with a total of 4–10 mice per group and tested with a two-tailed *t*-test (*p < 0.05).

**Figure 2:**
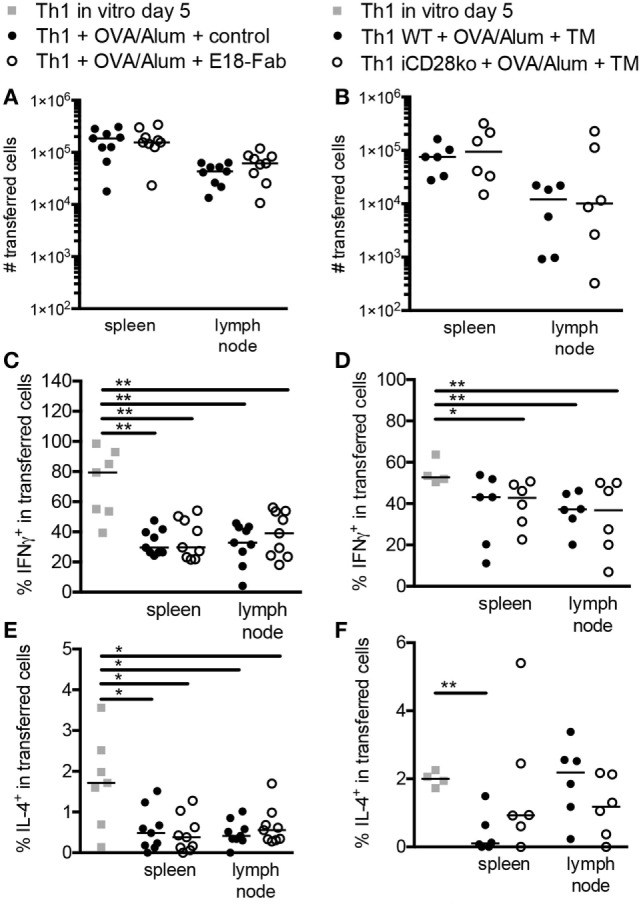
CD28 costimulation does not modulate differentiation of mouse T helper 1 cells *in vivo*. Additional analysis of the experiments presented in Figure 1. **(A)** Absolute cell numbers and medians of transferred cells 7 days after antigen administration with E18-Fab-mediated CD28 blockade or **(B)** inducible CD28 deletion. **(C,D)** Frequencies of interferon γ and **(E,F)** interleukin-4 producing cells among transferred cells 7 days after antigen administration in mice with **(C,E)** E18-Fab-mediated CD28 blockade or **(D,F)** tamoxifen-induced CD28 deletion. The data shown were pooled from three to five independent experiments with a total of 6–9 mice per group and tested with a two-tailed Mann–Whitney test (*p < 0.05, **p < 0.01, ***p < 0.001). Horizontal bars indicate medians per group.

In order to study the contribution of CD28 costimulation to Th1 recall responses, we first differentiated naive OT-II Thy1.1^+^ TCR-transgenic CD4^+^ T cells into Th1 cells *in vitro* before transferring them into C57BL/6 recipient mice (Thy1.2^+^) (Figures 1A,B). We then either blocked CD28–B7 ligand interactions with Fab fragments of mAb E18 (17, 18) or induced CD28 deletion by tamoxifen treatment of the recipient mice (Figures 1A,B). Afterward, we challenged the recipient mice with OVA/Alum and followed cytokine release in the serum for up to 48 h after the challenge (Figure 1C). Antigen challenge induced systemic IFNγ release into the circulation in mice which had received Th1 OT-II T cells (Figure 1C). Blocking CD28–B7 ligand interactions (Figure 1C, left graphs) or tamoxifen-induced CD28 deletion (Figure 1C, right graphs), however, clearly diminished IFNγ concentrations in the serum. This observation is in line with the known enhanced IFNγ expression during primary effector T cell responses after release of IFNγ mRNA from glyceraldehyde-3-hosphate dehydrogenase (GAPDH) upon induction of glycolysis (19), which itself is driven by CD28 costimulation (20). The reduction in IFNγ release was not accompanied by an induction of systemic IL-5 release, suggesting that there was no actual reprogramming of the Th1 cells toward a Th2 phenotype (8) (Figure 1C). Reduced overall cytokine release may be due to reduced secondary clonal expansion of the transferred Th1 cells in the absence of CD28 costimulation. Therefore, we quantified the yield of the progeny of the transferred Th1 cells retrieved 7 days after antigenic challenge *in vivo* (Figures 2A,B). Seven days post antigenic challenge is well within the phase of secondary (memory) CD4^+^ T cell expansion which has been shown to last until 30 days post antigenic challenge (21). Unlike to what has been described for naive T cells (1), CD28 costimulation was not critical for secondary clonal expansion of Th1 cells. Without antigenic challenge we were not able to detect any daughter cells of the transferred Th1 cells in the recipient mice (*n* = 4 mice analyzed) highlighting that the size of the OT-II Th1 cell pool at the time of analysis, indeed, reflected secondary clonal expansion. CD28 costimulation, thus, crucially enhanced cytokine release triggered by antigenic recall of Th1 cells *in vivo* without affecting secondary clonal expansion.

### CD28 Costimulation Does Not Modulate Lineage Differentiation of Mouse Th1 Cells *In Vivo*

Systemic IL-5 release (Figure 1C, lower panel) lacks sensitivity to be able to truly assess lineage differentiation and reprogramming of Th1 cells *in vivo*. We, therefore, re-analyzed IFNγ and IL-4 expression by intracellular FACS staining in Th1 cells 7 days after antigen challenge *in vivo* (Figures 2C–F). Compared to the Th1 cells analyzed at the end of the *in vitro* differentiation phase, there was a reduction in the frequencies of IFNγ-producing, and even the few IL-4-producing, cells after *in vivo* transfer and antigenic challenge. However, neither E18-Fab-mediated abrogation of CD28 signaling (Figures 2C,E) nor tamoxifen-induced CD28 deletion on the donor Th1 cells (Figures 2D,F) had an impact on the frequencies of IFNγ and/or IL-4 producers detected among the transferred Th1 cells after PMA/ionomycin restimulation. The data, thus, suggest that, despite clearly modulating systemic IFNγ release, CD28 costimulation had no impact on Th1 lineage stability *in vivo*.

### CD28 Costimulation Increases Cytokine Release From Th1 Cells *In Vitro*

**Figure 3:**
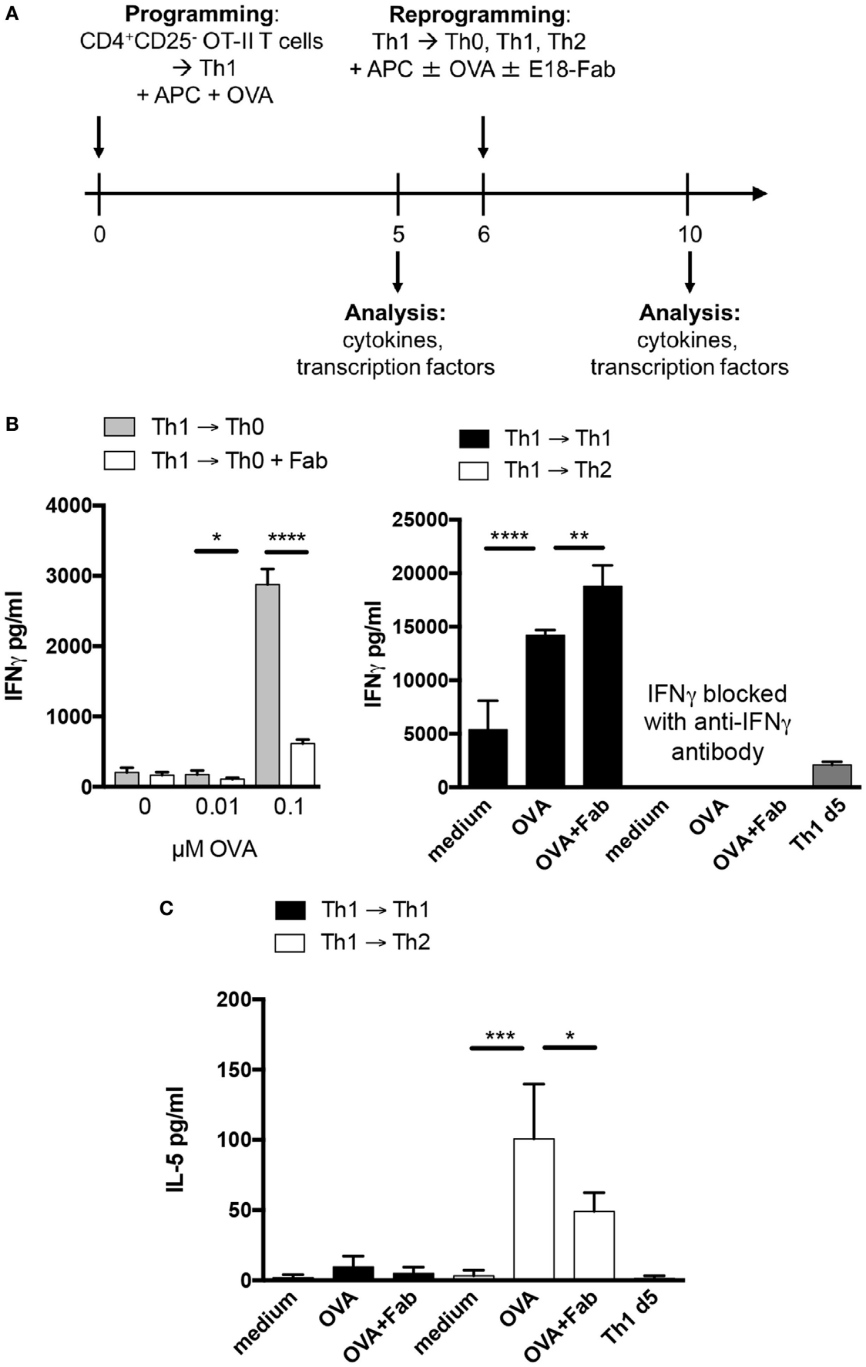
CD28 costimulation increases cytokine release from T helper 1 cells *in vitro*. **(A)** Isolated CD4^+^CD25^−^ T cells of OT-II Thy1.1^+/+^ mice were co-cultured with T cell-depleted splenocytes under Th1 conditions for 6 days and afterward reactivated under Th2, Th0, or Th1 conditions for additional 4 days in the presence and absence of OVA peptide and E18-Fab fragment. **(B)** Interferon γ concentrations in supernatants 4 days after restimulation under Th0 (left graph), Th1, or Th2 conditions (both right graph) in the presence or absence of E18-Fab and up to 0.1 µM (left) or 1 µM ovalbumin (OVA) peptide (right). **(C)** IL-5 concentrations in supernatants 4 days after restimulation with 1 µM OVA peptide under Th1 or Th2 conditions and in the presence or absence of E18-Fab. Graphs show mean + SD of triplicate cultures from one experiment. The results are representative of two to five independent experiments and were tested with a two-tailed unpaired *t*-test (*p < 0.05, **p < 0.005, ***p < 0.001, ****p < 0.0001).

Deletion of CD28 on the transferred Th1 cells was sufficient to reduce systemic IFNγ release *in vivo* (Figure 1C). The IFNγ measured in the serum might, however, in part have stemmed from bystander memory CD8^+^ T cells and NK cells fueled by the transferred Th1 cells, presumably through IL-2 secretion, to produce IFNγ (22). Therefore, we followed up on our *in vivo* data with a series of *in vitro* experiments allowing us to directly measure IFNγ release by the Th1 cells themselves, i.e., in the absence of memory CD8^+^ T cells (Figure 3A). In these *in vitro* experiments, we first determined the contribution of CD28 costimulation to IFNγ secretion by Th1 cells by restimulating them with different concentrations of antigen plus APCs and with or without addition of E18-Fab. IFNγ release from differentiated Th1 cells was dependent on CD28 costimulation (Figure 3B, left). After restimulation of the Th1 cells under Th1 conditions, the OT-II Th1 cells secreted very high amounts of IFNγ even without addition of antigen, i.e., OVA peptide (Figure 3B, right). IFNγ secretion was further enhanced upon addition of OVA peptide (1 µM) and even further increased in the presence of E18-Fab (Figure 3B, right). In contrast to restimulation under Th0 conditions (Figure 3B, left), under Th1 conditions CD28 costimulation, thus, did not enhance, but slightly reduced IFNγ secretion by Th1 cells (Figure 3B, right). As the absolute cell numbers of Th1 cells kept under Th1 conditions were not influenced by CD28 costimulation (data not shown) we could rule out that CD28-induced apoptosis of Th1 cells (23) accounted for this observation. IL-5 secretion, which was induced in Th1 cells transferred to Th2 conditions, was also reduced when CD28 costimulation was inhibited (Figure 3C). Taken together, IFNγ release from Th1 cells was clearly reduced upon inhibition of CD28-mediated costimulation *in vitro* unless the Th1 cells were restimulated under strongly pro-inflammatory Th1 conditions.

### CD28 Costimulation Drives Expansion of Human PPD-, but Not Td-Specific Memory T Helper Cells *In Vitro*

**Figure 4:**
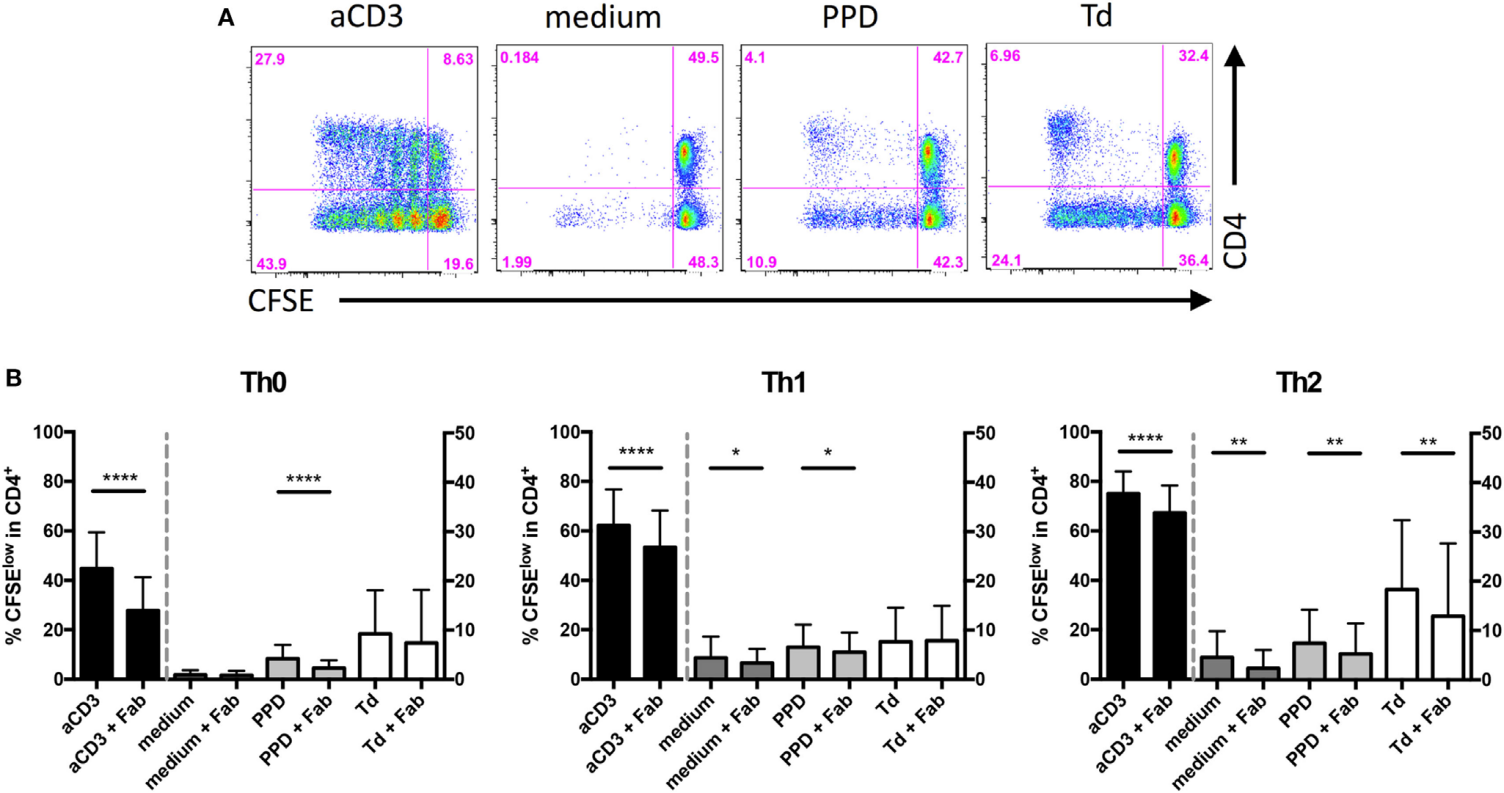
CD28 costimulation drives expansion of human purified protein derivative (PPD)-specific T helper 1 cells *in vitro*. Carboxyfluorescein succinimidyl ester diacetate (CFSE) (5 µM) labeled peripheral blood mononuclear cells were cultured in the presence or absence of anti-CD3 monoclonal antibody (mAb) (HIT3a), PPD, tetanus and diphtheria toxoid, and Fab fragments of anti-CD28 mAb CD28.3 under Th0, Th1, or Th2 conditions for 6 days. **(A)** Representative FACS data of CFSE dilution and CD4 expression. **(B)** Frequencies of proliferated cells among CD4^+^ cells cultured under Th0 (left), Th1 (middle), or Th2 (right) culture conditions. Graphs show mean + SD from 19 donors assayed individually and tested with a one-tailed paired *t*-test (*p < 0.05, **p < 0.005, ****p < 0.0001).

**Figure 5:**
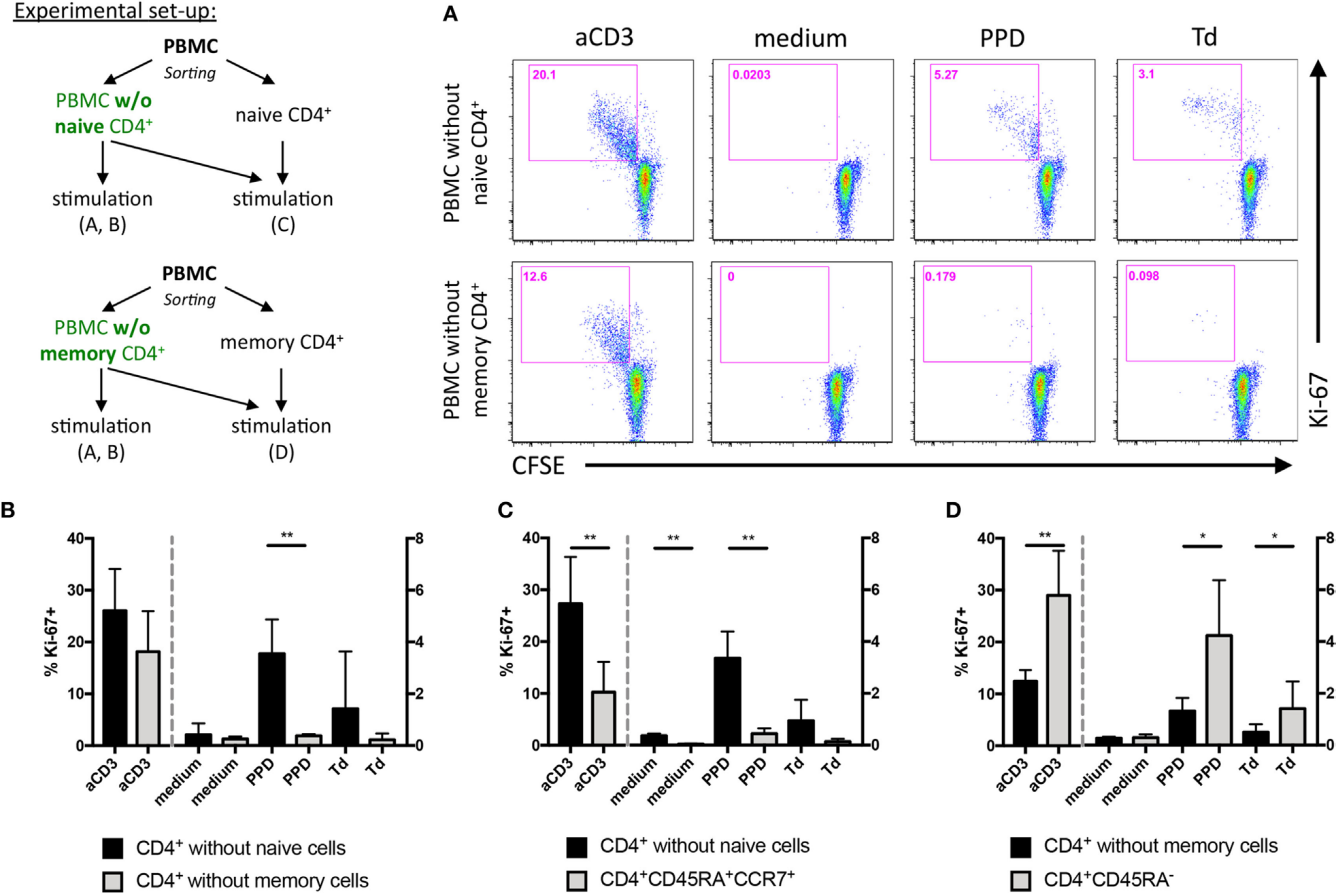
Stimulation of human peripheral blood mononuclear cells (PBMC) with purified protein derivative or tetanus and diphtheria toxoid induces proliferation of memory CD4^+^ T cells *in vitro*. PBMC were depleted of naive or memory CD4^+^ T cells by flow cytometric cell sorting and labeled with carboxyfluorescein succinimidyl ester diacetate (CFSE). As summarized in the scheme, the CFSE-labeled PBMC were then either stimulated alone or with addition of (unlabeled) naive or memory CD4^+^ T cells. **(A)** CFSE dilution among memory (upper row) or naive CD4^+^ T cells (lower row) and Ki-67 expression were analyzed after 4 days of culture in the presence of the indicated stimuli. **(B)** Summary graph of experiments set up as in **(A)** showing frequencies of Ki-67^+^ cells among CD4^+^ T cells from three independent experiments with cells from one donor each. **(C)** Frequencies of Ki-67^+^ cells among CD4^+^ CD45RA^+^ CCR7^+^ naive T cells (gray columns) added back to naive CD4^+^ T cell-depleted PBMC (black columns: % Ki-67^+^/memory CD4^+^ T cells). **(D)** Reverse experiment of **(C)**, i.e., frequencies of Ki-67^+^ cells among CD4^+^ CD45RA^−^ memory T cells (gray columns) added back to memory CD4^+^ T cell-depleted PBMC (black columns: % Ki-67^+^/naive CD4^+^ T cells). Columns are mean + SD of values from three donors assayed individually and tested with a one-tailed paired *t*-test (*p < 0.05, **p < 0.005).

As our data obtained in mice *in vivo* and with mouse Th1 cells *in vitro* showed that CD28 costimulation enhanced IFNγ release by Th1 cells we tested whether *in vivo* differentiated pathogen-specific human memory T cells would behave similarly. In mice, we had observed that Th1 cell expansion *in vivo* was not affected by CD28 costimulation (Figures 2A,B) and also *in vitro* CD28 costimulation had no effect on the expansion of Th1 cells (data not shown). To test the impact of CD28 costimulation on the expansion of human CD4^+^ T cells *in vitro* under Th0, Th1, and Th2 conditions, we stimulated human PBMC either with an anti-CD3 mAb (clone HIT3a), the recall antigen PPD produced by mycobacteria or Td from *Clostridium tetani* and *Corynebacterium diphtheriae*. While Td-specific CD4^+^ T cells comprise both Th1 and Th2 cells (24–26), PPD-specific CD4^+^ T cells are predominantly of a Th1 phenotype (27). To inhibit CD28 costimulation we used Fab fragments of the anti-human CD28 mAb CD28.3 (28) at 0.3 µg/ml, which we had determined to be optimal to inhibit anti-CD3 mAb-induced proliferation of CD4^+^ T cells within PBMC (data not shown). To determine the contribution of CD28 costimulation to the expansion of human T cells in the PBMC cultures we analyzed the percentage of CFSE^low^ cells among CD4^+^ T cells at the end of the culturing period on day 6 (Figures 4A,B). Blocking CD28 costimulation reduced the yield of CFSE^low^ cells among CD4^+^ T cells after addition of either anti-CD3 mAb (Figure 4B, black columns) or PPD (Figure 4B, light gray columns) independently of the cytokine milieu. In contrast, the expansion of Td-specific CD4^+^ T cells was only modulated by CD28 costimulation under Th2 conditions (Figure 4B, white columns). To verify that the CD4^+^ T cells responding to antigenic recall were, indeed, memory cells we depleted PBMC of either CD45RA^−^ memory or CD45RA^+^ CCR7^+^ naive CD4^+^ T cells by flow cytometric cell sorting. After depletion of naive CD4^+^ T cells, recall responses to PPD and Td could still be detected, which was not the case after depletion of memory CD4^+^ T cells (Figures 5A,B). In parallel cultures, we added back the previously depleted cells and then compared cell proliferation by analyzing Ki-67 expression of naive and memory CD4^+^ T cells which were either CFSE-labeled or unlabeled (Figures 5C,D). To ensure that we would be able to clearly detect Ki-67 expression we analyzed all the cultures in Figure 5 already after 4 days instead of 6. Even in the presence of memory CD4^+^ T cells there was minimal bystander proliferation of naive CD4^+^ T cells toward recall antigens. Moreover, the memory CD4^+^ T cells responded well toward PPD and Td stimulation independently of whether they had been labeled with CFSE or not. When naive and memory CD4^+^ T cells were co-cultured, the naive CD4^+^ T cells even showed a clearly reduced response toward anti-CD3 stimulation (Figures 5C,D) suggesting that also in unseparated PBMC the CD4^+^ T cell response to anti-CD3 mAb stimulation was mainly due to responding memory cells (Figure 4).

Together, expansion of anti-CD3 mAb stimulated total human CD4^+^ T cells as well as PPD-specific memory CD4^+^ T cells was enhanced by CD28 costimulation. In contrast, expansion of Td-specific memory CD4^+^ T cells was largely independent of CD28 costimulation.

### CD28 Costimulation Favors GATA-3 Over T-Bet Expression in Human Memory CD4^+^ T Cells

**Figure 6:**
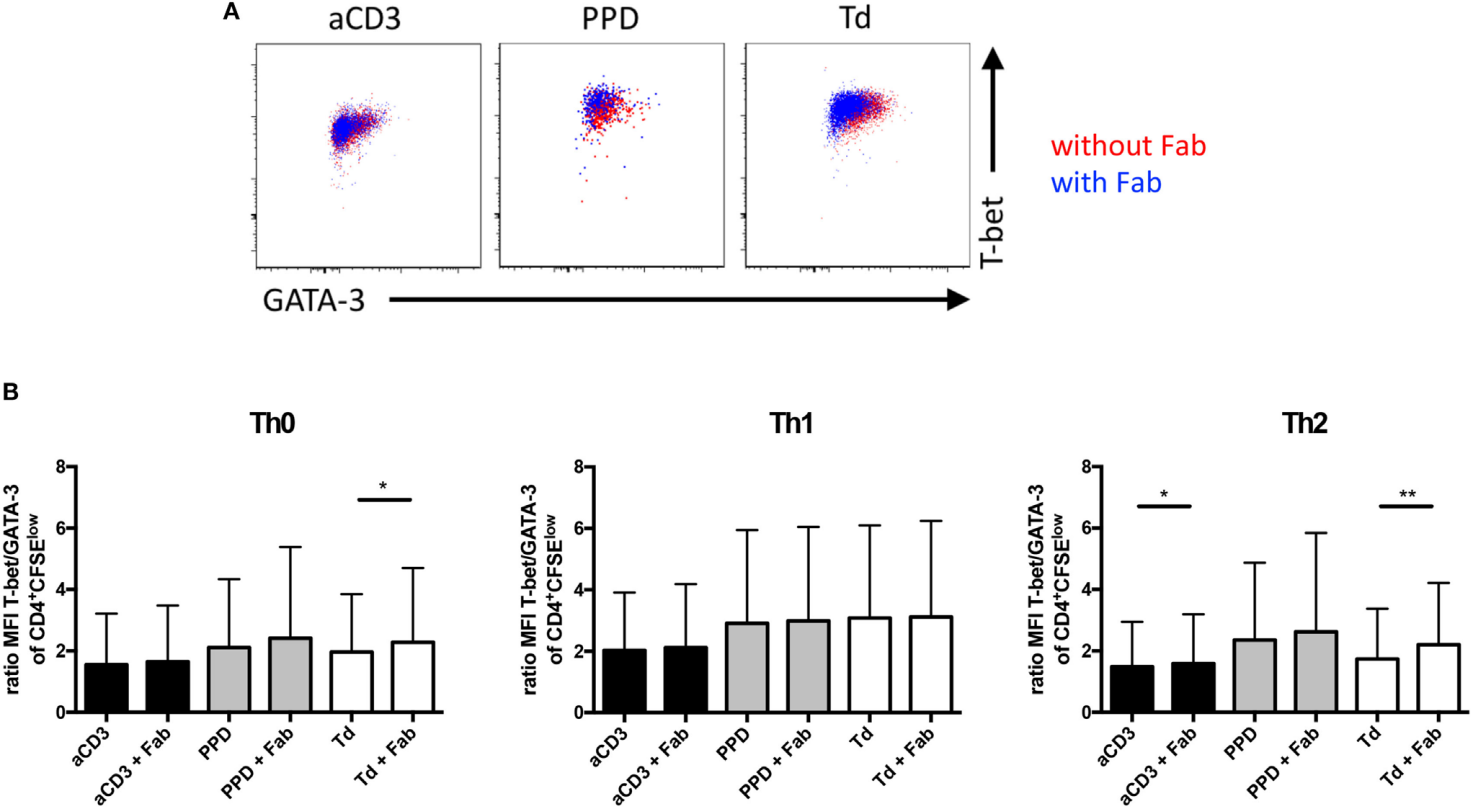
CD28 co-stimulation favors GATA-3 over T-bet expression in tetanus and diphtheria toxoid-specific human memory CD4^+^ T cells upon antigenic recall under Th2 conditions *in vitro*. Additional analysis of experiments presented in Figure 4. **(A)** Representative FACS data of GATA-3 and T-bet expression in CD4^+^ T cells cultured in the absence (red) or presence (blue) of CD28.3-Fab. **(B)** Normalized ratio of median fluorescence intensity (MFI) T-bet/MFI GATA-3 in proliferated CD4^+^carboxyfluorescein succinimidyl ester diacetate^low^ cells cultured under Th0 (left), Th1 (middle), or Th2 (right) culture conditions. Graphs show mean + SD from 19 donors assayed individually and tested with a one-tailed paired *t*-test (*p < 0.05, **p < 0.005).

Antigenic stimulation of CFSE-labeled human PBMC with PPD or Td allowed us to identify antigen-reactive memory CD4^+^ T cells (Figure 4A). Therefore, we could assess how CD28 costimulation affects T-bet and GATA-3 expression in human memory CD4^+^ T cells as markers of lineage stability. Under Th0 conditions blockade of CD28–ligand binding increased the T-bet/GATA-3 ratio in Td-specific memory CD4^+^ T cells (Figures 6A,B—left graph, white columns). The same was true for Td-specific cells stimulated in a Th2 milieu (Figure 6B—right graph, white columns). Pro-inflammatory Th1 polarizing conditions, however, *per se* induced a higher T-bet/GATA-3 ratio in PPD- and Td-specific memory CD4^+^ T cells than Th0 conditions (Figure 6B—middle graph, gray, and white columns), which was not modulated by blockade of CD28 costimulation. In anti-CD3 mAb-stimulated CD4^+^ T cells inhibition of CD28–ligand binding led to a higher T-bet/Gata3 ratio under Th2 conditions (Figure 6B—black columns). The differentiation status of human, particularly Td-specific, memory CD4^+^ T cells was, thus, sensitive toward CD28 costimulation, which favored reprogramming toward a Th2 phenotype characterized by a low ratio of T-bet/GATA-3 expression.

### IFNγ and IL-5 Release From Human PBMC Is Enhanced by CD28-Mediated Costimulation

**Figure 7:**
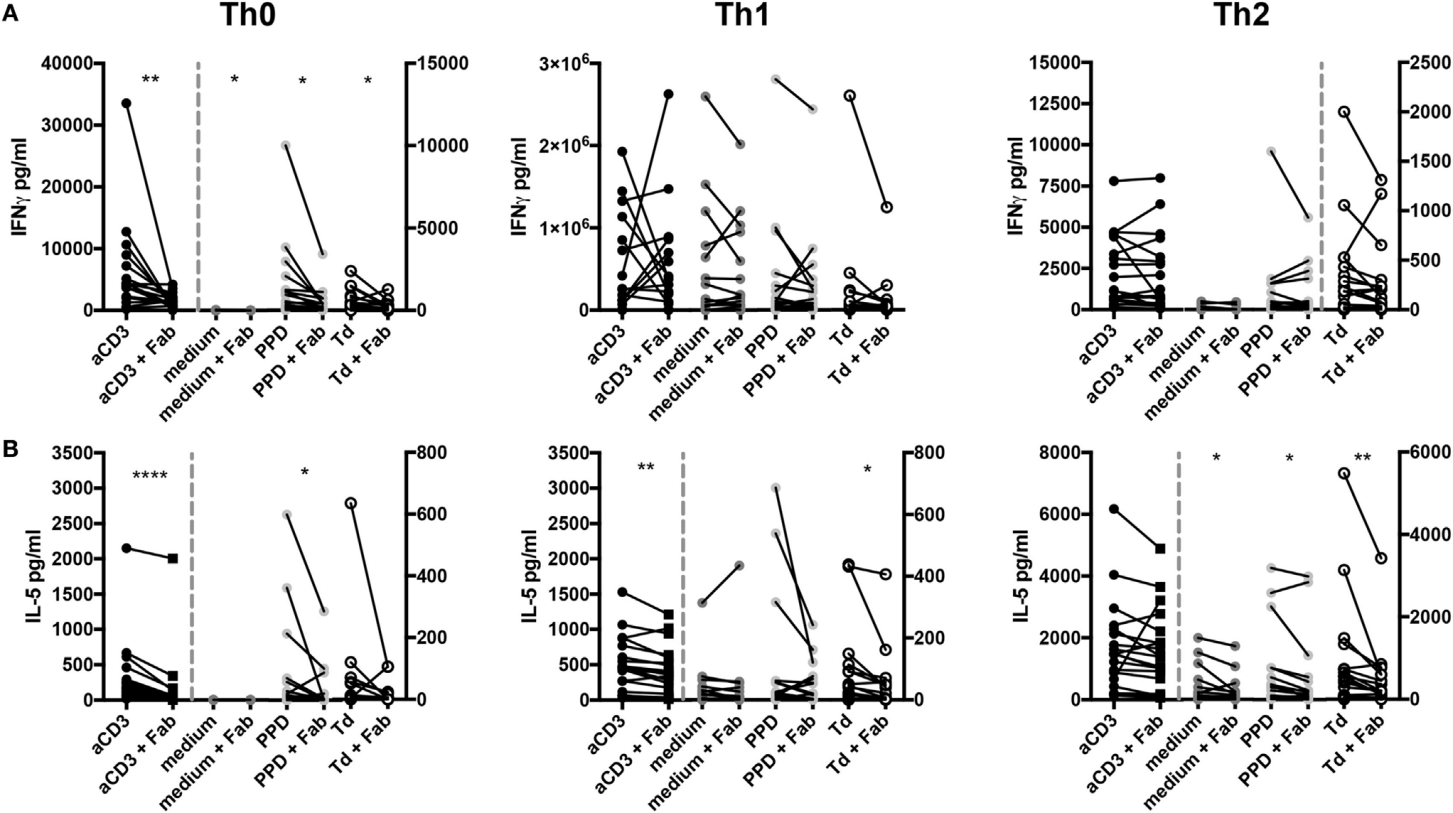
CD28 costimulation enhances cytokine release from peripheral blood mononuclear cells (PBMCs) upon antigenic recall stimulation with purified protein derivative or tetanus and diphtheria toxoid *in vitro*. **(A)** Interferon γ (IFNγ) and **(B)** interleukin-5 concentrations in supernatants of experiments are first shown in Figure 4, i.e., under Th0 (left), T helper (Th1) (middle), or Th2 (right) culture conditions. Please note that for human T cells Th1 conditions did not comprise addition of an anti-IFNγ antibody as was the case for mouse Th1 conditions. Graphs show data from 19 donors assayed individually and tested with a one-tailed paired *t*-test (*p < 0.05, **p < 0.005).

Challenging mice with antigen *in vivo* (Figure 1) and culturing mouse Th1 cells *in vitro* (Figure 3) showed that CD28 costimulation enhanced IFNγ release from Th1 cells. Therefore, we tested whether CD28 costimulation would also modulate cytokine release from human PBMC stimulated with PPD or Td and, for comparison, anti-CD3 mAb in solution. We observed that IFNγ release triggered by anti-CD3 mAb, PPD, or Td was enhanced upon CD28 costimulation when the cells were kept under Th0 conditions (Figure 7A, left graph). Shifting the culture conditions to pro-inflammatory Th1 abolished the need for CD28 costimulation to obtain maximum IFNγ release (Figure 7A, middle graph). Under Th2 conditions, in which human T cells did not comprise addition of an anti-IFNγ mAb, the amounts of IFNγ detectable in the supernatants were reduced compared to Th0 and Th1 conditions. Here, CD28 costimulation also did not enhance IFNγ release (Figure 7A, right graph). IL-5 secretion was enhanced by CD28-mediated costimulation under Th0 conditions upon stimulation with anti-CD3 mAb or PPD (Figure 7B, left graph). Under Th2 conditions CD28 costimulation increased IL-5 secretion upon addition of PPD or Td (Figure 7B, right graph), while in the Th1 milieu IL-5 release was enhanced by CD28 costimulation together with anti-CD3 mAb or Td (Figure 7B, middle graph). CD28 costimulation, thus, enhanced secretion of IFNγ and IL-5 from human PBMC upon antigenic recall stimulation.

## Discussion

In this study, we addressed the role of CD28 costimulation for mouse Th1 T cell function both *in vivo* and *in vitro*. Our data identify CD28 costimulation as a key driver of IFNγ secretion induced by restimulated Th1 cells *in vivo*. This positive effect of CD28 costimulation was not restricted to mouse T cells as cytokine secretion by human PBMC stimulated with T cell recall antigens *in vitro* was also enhanced by CD28 costimulation.

We first differentiated OVA-specific OT-II CD4^+^ T cells into Th1 cells *in vitro* before adoptive transfer into fully immunocompetent C57BL/6 recipient mice. In contrast to many previous studies addressing the role of CD28 costimulation for effector and memory CD4^+^ T cell function, it was only then that we interfered with CD28 costimulation by specific targeting. We used either Fab fragments of the anti-CD28 mAb E18 blocking ligand binding to CD28 (18) or we genetically deleted CD28 expression in iCD28ko OT-II Th1 cells. This strategy avoids the pitfalls associated with the use of CTLA-4-Ig and with CD28 deletion already during initial clonal expansion (3, 6). In our study, CD28-deficiency was truly confined to the period of antigenic restimulation *in vivo*, a situation coming very close to clinical scenarios in humans who generally harbor huge populations of fully differentiated (pathological) T cells by the time patients seek medical attention.

CD28 costimulation had no impact on the secondary expansion of the Th1 cells *in vivo* (Figures 2A,B), which is, of course, in contrast to primary responses of naive CD4^+^ T cells (1). We, however, noted that the Th1 cells underwent a certain degree of reprogramming toward a Th2 phenotype *in vivo* as indicated by reduced frequencies of IFNγ producers and a lower ratio of the MFIs for T-bet and Gata-3 in the recovered OT-II T cells (Figure 2 and data not shown). This reprogramming most likely reflects the change in milieu the OT-II T cells experience after transfer from the strongly Th1-polarizing *in vitro* cultures into healthy C57BL/6 recipient mice, i.e., into animals without an ongoing immune response.

In contrast to secondary clonal expansion and lineage stability, IFNγ release was markedly reduced upon CD28 blockade *in vitro* (Figure 3) and *in vivo* (Figure 1C left graph) or after genetic deletion of CD28 on Th1 cells *in vivo* (Figure 1C right graph). As genetic CD28 deletion only affected CD28 expression by the transferred Th1 OT-II T cells, but not bystander T cells, it was CD28 costimulation of the transferred Th1 cells themselves, which controlled systemic IFNγ release. Our *in vitro* restimulation data further confirmed that IFNγ release from Th1 cells was enhanced upon CD28 costimulation (Figure 3B). *In vivo*, however, we assume that IFNγ secretion by bystander memory CD8^+^ T cells (22), after activation by the transferred Th1 cells, contributed to the total amount of IFNγ released into the circulation. This further means that the amount of IFNγ released upon costimulation of Th1 cells *in vivo* should positively correlate with the size of the memory CD8^+^ T cell compartment in an animal. We would, thus, expect that mice kept under “pet shop-like” conditions with similarly high immunological competence as adult humans (29) would show even stronger CD28-dependent IFNγ release upon Th1 cell recall stimulation than the cleanly housed laboratory animals we used in our study. For the release of cytokines from human memory T cells this, in turn, means that the large memory compartment in peripheral blood T cells should allow to determine the impact of CD28 costimulation on cytokine secretion upon antigenic recall with high sensitivity.

Studying human T cell responses to anti-CD3 mAb stimulation and recall responses to PPD and Td we observed that the CD28.3-Fab reduced the expansion of anti-CD3 mAb- and PPD-stimulated human CD4^+^ T cells (Figure 4), which is in accordance with previous studies using FR104 to inhibit expansion of alloreactive and viral peptide-specific human T cells (10, 11). Expansion of Td-specific CD4^+^ T cells was, however, not affected by blocking CD28 signaling (Figure 4). This might be due to the polarization of the Td-specific CD4^+^ T cells comprising both Th1 and Th2 cells (24–26), while viral antigen- (30–33) and PPD-specific T cells (27) in healthy subjects are predominantly of a Th1 phenotype. Moreover, memory B cells specific for PPD (34) or Td (35, 36), of course, readily take up antigen and present it to the memory T cells. Therefore, the pool of APCs differs between these bacterial recall antigens studied here and viral peptides loaded onto HLA molecules externally (10). The contribution of CD28 costimulation to secondary expansion of human memory T cells with different antigenic specificities, thus, varies, which is probably due to the composition of the different memory T cell pools (Th1/Th2) and to the type of cell presenting the antigen.

CD28 costimulation shifted Td-specific memory T cells toward a Th2 phenotype with regards to GATA-3 and T-bet expression (Figure 6). CD28 costimulation, thus, appears to not only favor GATA-3 over T-bet expression in naive (mouse) CD4^+^ T cells (37), but also in human memory T cells. In naive CD4^+^ T cells CD28 costimulation has been shown to enhance (38–41) and, under certain conditions, to be even sufficient to induce Th2 differentiation (42). In contrast, CD28 costimulation of mouse memory CD4^+^ T cells *in vivo* (Figure 1) and human memory CD4^+^ T cells *in vitro* (Figure 7) did not actually reprogram the cells toward a Th2 phenotype marked by high IL-4 or IL-5 and low IFNγ secretion. A lower T-bet/GATA-3 ratio may, however, have an impact on T cell migration given the distinctive expression of chemokine receptors on human Th1 and Th2 T cells (43, 44), which we did not study here, but which could, of course, substantially contribute to modulation of memory T cell responses *in vivo*. The reason we did not include chemokine receptor expression as a means to define human Th cell subsets in our study was that Ficoll density centrifugation of the cells obtained from the LRS-C led to a very high degree of internalization of chemokine receptors (45) so that these were undetectable on the cell surface by flow cytometry (data not shown). Also in line with published work (45) activation of the T cells *in vitro* did not induce re-expression of the receptors, i.e., after 6 days of culture there was no differential expression of chemokine receptors on the cultured T cells (data not shown).

A key result of our experiments with human PBMC was that CD28 costimulation substantially enhanced IFNγ secretion after stimulation with anti-CD3 mAb, PPD, or Td (Figure 7). Control of cytokine release was, however, not restricted to IFNγ as IL-5 secretion upon PPD stimulation under Th0 conditions and upon PPD or Td stimulation under Th2 conditions was also markedly reduced upon CD28 inhibition (Figure 7)—as was the case for mouse OT-II Th1 cells cultured under Th2 conditions (Figure 3C).

Molecularly, we assume that induction of glycolysis and increasing mitochondrial respiratory capacity by CD28 costimulation (20, 46) in the memory T cells enhances both IFNγ (19) and IL-5 as well as GATA-3 expression (47). IFNγ production by Th1 cells relies on GAPDH being recruited to glycolytic processes thus liberating IFNγ mRNA (19, 48). Regarding proliferation, CD4^+^ T cells may cover their energy supply either by glycolysis or oxidative phosphorylation (19). These two observations together could explain why secondary expansion of the OT-II Th1 cells *in vivo* was not impaired in the absence of CD28 costimulation (Figure 1), while systemic IFNγ release was reduced (Figure 1). Moreover, the reduced dependence of CD4^+^ T cells, compared to CD8^+^ T cells, on glycolysis for activation-induced expansion (49, 50) also explains why memory CD8^+^ T cells depend more strongly on CD28 costimulation for optimal expansion upon antigenic recall *in vivo* (51) than CD4^+^ T cells.

Finally, our observation that CD28 costimulation plays a key role in CD4^+^ T cell recall responses also provides further explanations as to why it is biologically reasonable to target the CD28 pathway by the inhibitory receptors CTLA-4 (52) and PD-1 (53, 54) which themselves are only expressed after T cell activation. Upon therapeutic blockade of PD-1 on CD8^+^ T cells it has been shown that CD28 signaling is liberated allowing for full memory T cell responses to occur (54). Moreover, our data suggest that in situations of continued auto-aggression by memory CD4^+^, and probably also CD8^+^, T cells, therapeutic inhibition of CD28 costimulation might be efficacious. This is, for example, the case in patients suffering from multiple sclerosis who harbor myelin-reactive Th1 cells (55). Further, blocking CD28 ligation may also facilitate actual reprogramming of pathogenic Th1 cells toward, e.g., a Th2 phenotype. Reprogramming of both mouse (8) and human CD4^+^ Th cells (7) requires TCR stimulation and an appropriate cytokine milieu. TCR ligation, however, comes with the risk of inducing an (initial) flare of the disease (55), which, according to our data, might be avoided by concomitant inhibition of CD28 ligation by co-treatment of patients with the anti-CD28 Fab’ antibody fragment FR104 (10, 11).

## Ethics Statement

All animal experiments were performed in accordance with German law and approved by the Regierung von Unterfranken as the responsible authority. The ethics committee of the medical faculty of the University of Würzburg approved the anonymous use of human PBMC from healthy blood donors for this study.

## Author Contributions

DL and NB designed the experiments. DL, SH, SG, AU, FL, BV, and TH contributed to performing and analyzing the experiments and interpreting the results. NB directed the study and wrote the manuscript, with input from DL, SH, and TH.

## Conflict of Interest Statement

BV is chief operating officer and shareholder of OSE immunotherapeutics. All other authors have declared that no conflict of interest exists.

## Abbreviations

APC, antigen-presenting cell; CFSE, carboxyfluorescein succinimidyl ester diacetate; GAPDH, glyceraldehyde-3-hosphate dehydrogenase; iCD28ko, inducible CD28 knock-out; IFNγ, interferon γ; IL, interleukin; mAb, monoclonal antibody; MFI, median fluorescence intensity; MHC, major histocompatibility complex; OVA, ovalbumin; PBMC, peripheral blood mononuclear cells; PPD, purified protein derivative; Td, tetanus and diphtheria toxoid; Th1 cells, CD4^+^ T helper 1 cells.
